# Generation and Role of Reactive Oxygen and Nitrogen Species Induced by Plasma, Lasers, Chemical Agents, and Other Systems in Dentistry

**DOI:** 10.1155/2017/7542540

**Published:** 2017-10-24

**Authors:** Nayansi Jha, Jae Jun Ryu, Eun Ha Choi, Nagendra Kumar Kaushik

**Affiliations:** ^1^Department of Oral and Maxillofacial Implantology, Graduate School of Clinical Dentistry, Korea University, Seoul, Republic of Korea; ^2^Plasma Bioscience Research Centre, Department of Electrical and Biological Physics, Kwangwoon University, Seoul, Republic of Korea

## Abstract

The generation of reactive oxygen and nitrogen species (RONS) has been found to occur during inflammatory procedures, during cell ischemia, and in various crucial developmental processes such as cell differentiation and along cell signaling pathways. The most common sources of intracellular RONS are the mitochondrial electron transport system, NADH oxidase, and cytochrome P450. In this review, we analyzed the extracellular and intracellular sources of reactive species, their cell signaling pathways, the mechanisms of action, and their positive and negative effects in the dental field. In dentistry, ROS can be found—in lasers, photosensitizers, bleaching agents, cold plasma, and even resin cements, all of which contribute to the generation and prevalence of ROS. Nonthermal plasma has been used as a source of ROS for biomedical applications and has the potential for use with dental stem cells as well. There are different types of dental stem cells, but their therapeutic use remains largely untapped, with the focus currently on only periodontal ligament stem cells. More research is necessary in this area, including studies about ROS mechanisms with dental cells, along with the utilization of reactive species in redox medicine. Such studies will help to provide successful treatment modalities for various diseases.

## 1. Introduction

Reactive oxygen and nitrogen species (RONS) are free radicals and reactive molecules derived from molecular oxygen and nitrogen species. These can act as intercellular as well as intracellular messengers. Those most commonly generated ones are hydroxyl radicals (^•^OH), hydrogen peroxide, nitric oxide, peroxides, peroxynitrite, singlet oxygen, and superoxides [1].

In this review, we discuss about the types of reactive oxygen species (ROS, intracellular and extracellular) and their roles in cell signaling pathways with a focus on the sources of ROS in the dental field. The roles of nonthermal plasma as a source of these reactive species and their therapeutic applications are also discussed. A section about the effects of reactive species on dental cells and their role in dentistry is also included.

### 1.1. Intracellular ROS

RONS are generated in biological systems as metabolic byproducts as well as signaling molecules. ROS as a signaling molecule was studied in sea urchins by Wong et al. [2]. It was found that to block polyspermy in sea urchins, biological transformation of the egg cellular matrix could be done, resulting in the formation of a fertilization envelope (solid structure). For this process, the enzyme ovoperoxidase had to be targeted towards the fertilization envelope. The level of ovoperoxidase was found to be increased due to increased levels of one of the substrates, −H_2_O_2_. The increase in H_2_O_2_ is a calcium-dependent mechanism involving oxidase activity [2].

**Figure 1:**
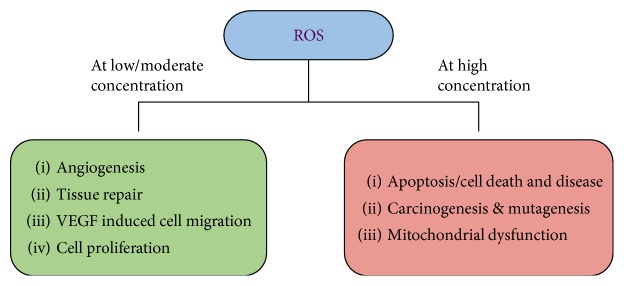
ROS activity within mammalian cells. At low levels, positive effects such as tissue repair and cell differentiation are initiated, while at high levels, uncontrolled cell activity may result in mitochondrial dysfunction, mutagenesis, and apoptosis.

Free radicals are mostly generated from oxygen, like superoxide anions (O_2_^•−^), hydroxyl ions (OH^−^), hydrogen peroxide (H_2_O_2_), nitric oxide (NO), and hydroxyl radicals (^•^OH) [3]. ROS at low levels are beneficial for cells, with increased growth activity, tissue repair, cell proliferation, and angiogenesis [4] whereas at very high unregulated levels, deleterious effects may arise, leading to cell death [5] and apoptosis (Figure 1).

ROS are present everywhere; they are produced as metabolic byproducts in numerous diseases, especially those associated with inflammation, injury, and cell ischemia [6]. Even inflammation of the dental pulp can cause a release of these reactive particles. ROS generation may be pathological or physiological and is dependent on various circumstances. The mitochondrial electron transport system and NADPH oxidase are the most common sources of ROS, while cytochrome P450 and uncoupled endothelial nitric oxide synthase (eNOS) [5] as well as myeloperoxidases are some of the other sources that can generate these reactive free radicals [3].

**Figure 2:**
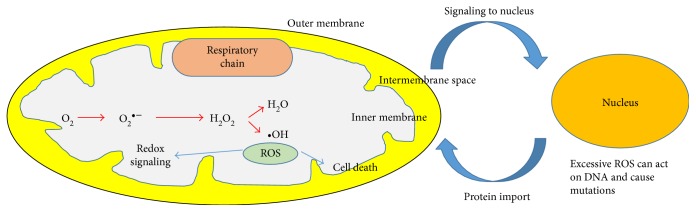
Redox reactions within cells. Mitochondrial cell produces hydrogen peroxide, which is formed by the dismutation of O2^•−^ within the mitochondria. The production of free radicals within the mitochondria may occur in the outer membrane, in the inner membrane, or within the matrix. Mitochondrion and nucleus interactions occur; however, excessive ROS can cause mutations due to DNA damage.

The mitochondria are the main source of ROS in mammalian cells. The production of ROS within mitochondria can occur in the outer membrane, in the inner membrane, or within the matrix. Isolated mitochondria produce hydrogen peroxide, which is formed by the dismutation of O_2_^•−^ within the mitochondria (Figure 2). This production of O_2_^•−^ occurs when there is a buildup of NADH or when there is a reduced CoQ pool within the mitochondria [7]. The entire process of O_2_^•−^ generation is highly complex and involves thermodynamic reactions within the mitochondria [7]. There are various redox centres in the mitochondrial electron transport chain which can result in electron leakage to oxygen, making it the primary superoxide source in most tissues [8].

**Figure 3:**
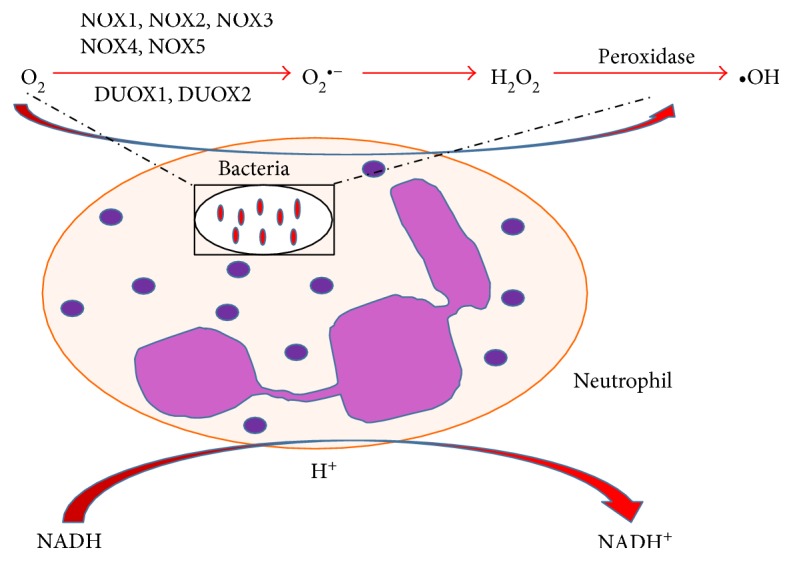
Free radical generation in neutrophils and macrophages during phagocytosis. Reactive species are released during phagocytosis. NADPH oxidases/NOX enzymes (NOX1, NOX2, NOX3, NOX4, and NOX5) and dual oxidases (DUOX1, DUOX2) facilitate the conversion of oxygen to superoxides using NADPH as an electron donor with ROS released as secondary products.

NADH is a nonmitochondrial, enzymatic source of reactive species found during phagocytosis in macrophages and neutrophils. There are a group of NOX (NADPH oxidases) enzymes that facilitate the conversion of oxygen to superoxide on biological membranes using NADPH as an electron donor with ROS released as secondary products [3] (Figure 3).

Host defense is a primary function of the NOX family, facilitating the killing of microorganisms by the release of ROS. They are also known to play a role in cellular signaling and induce a calcium release from intracellular stores. NOX-dependent ROS can also result in the regulation of gene expressions such as that associated with TNF-alpha [9]. Purified endothelial nitric oxide synthase can also generate superoxide during the process of cell signaling using nitric oxide (NO) [10]. NO^•^ produced by eNOS and inducible NOS (iNOS) interact with O_2_^•^ and produce peroxynitrite (ONOO^•^), which is harmful to vascular cells and which may also be involved in tumorigenesis [11].

The direct killing of microorganisms is not solely done by oxidants, as other mechanisms such as phagocytosis and the release of antimicrobial products also facilitate this process. Inside phagosomes, bacteria are ingested and killed by neutrophils. This process is accompanied by the action of NADPH oxidase, (which converts oxygen to superoxide). Cytoplasmic granules, for example, myeloperoxidases, are also released within these phagosomes, forming strong acids (with a decrease in pH) and causing microbial destruction [12].

Other sources of intracellular ROS are the cytochrome P450 enzymes. The cytochrome P450 enzymes (CYP), present in the liver, produce free radicals as a result of metabolizing xenobiotics (crucial in endogenous functions). CYP enzymes convert some xenobiotics into toxic quinones and semiquinones, which generate H_2_O_2_ and superoxide anions [13]. Cytochrome P450 enzymes (heme-thiolate enzymes) are also responsible for the oxidation of lipophilic compounds. If there is poor coupling of the P450 catalytic cycle, it can cause the continuous production of ROS. This continuous generation can cause lipid peroxidation, cell toxicity, and death [14].

However, various antioxidant systems such as catalases, GSH-Px (glutathione peroxidases), and the SODs (superoxide dismutases) are present to counteract the effect of elevated ROS levels and are activated under oxidative stress [15]. SODs are not an antioxidant per se, as they remove the superoxide, but they cause the generation of hydrogen peroxide. The H_2_O_2_ thus generated is further reduced to water by catalase and GSH-Px (glutathione) [16].

### 1.2. ROS and Cell Signaling Pathways

It has been found in various studies before that ROS have an impact on signaling pathways. As a result of the interaction of reactive species with various signaling molecules (due to oxidative stress or reduction), a number of processes, such as differentiation, iron hemostasis, and DNA and nucleic acid cycles, are affected [17] (Table 1).

**Figure 4:**
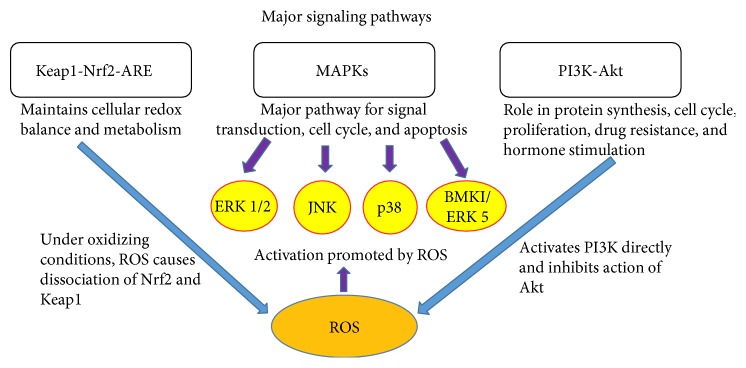
ROS and major signaling pathways. ROS activates signaling molecules within various pathways, such as MAPKs (mitogen-activated protein kinase) (the major pathway for cell cycles and apoptosis), Keap-1-Nrf2-ARE (a regulator of the cellular redox balance and metabolism), and PI3K-Akt (a regulator of protein synthesis, cell proliferation, and drug resistance). MAPKs = mitogen-activated protein kinases, ERK = extracellular signal-regulated kinases, and JNK = c-Jun N-terminal kinases.

Increase in ROS levels activates and promotes signaling molecules within various pathways, such as mitogen-activated protein kinase (MAPKs), Keap-1-Nrf2-ARE, and PI3K-Akt. MAPKs are protein kinases involved in a variety of cellular functions which are activated by ROS, but the mechanism of action is unclear [18]. There may be modifications in the amino acid sequence of related proteins, resulting in the activation of MAPKs, or certain oxidative changes in intracellular kinases. The Keap-1-Nrf2-ARE pathway is important for maintaining the cellular redox, balance, and metabolism. Under oxidizing conditions, an increase in ROS causes a dissociation between Nrf2 and Keap-1 (using Cys 151, Cys 288, and Cys 273). For the PI3K-Akt pathway, ROS activates PI3k directly and inhibits the activation of Akt. At low levels of Akt, ROS can be removed from the cells, with normal growth then taking place [19] (Figure 4).

## 2. Sources of ROS in Dentistry

Reactive oxygen species are known to have effects on wound healing, immunological response generation, and antibacterial properties, all of which have made them popular during dental treatments. However, ROS may also be a byproduct of resin cements, photosensitizers, and lasers, used often in dentistry, which may be harmful to cell survival in the long run (Table 2).

### 2.1. Nonthermal Atmospheric Pressure Plasma Applications

Plasma medicine has emerged as a field of research combining the aspects of physics and life sciences [20, 21]. Cold atmospheric pressure plasma (CAP) has been used extensively for biomedical applications. Miyamoto et al. [22] reported that the formation of a blood clot by low-temperature plasma treatment was faster than that by natural coagulation. Utsumi et al. [23] studied the effect of plasma on chemoresistant ovarian cancer cells. They demonstrated that plasma-activated media (PAM) can have antitumor effects on chemoresistant cells both in vitro and in vivo. Fathollah et al. [24] showed that plasma therapy is beneficial for diabetic wound healing in rats. They noted accelerated cell proliferation and keratinocyte migration. These findings may be beneficial for future treatments of diabetes mellitus. Studies are being done to identify plasma applications in microsurgery. Kieft et al. [25] cultured ovarian cells (CHO-K1) and found that at very low applied power levels (0.1–0.2 W), detachment of the cells from the surface occurred. At higher power levels (>0.2 W), cell necrosis was found to occur. Such evaluations can be utilized in future studies of cell manipulation in tissues [25].

The plasma needle has also emerged as a nonaggressive plasma source which can be used on mammalian cells and tissues without damaging the cells or causing cell necrosis. Nonthermal plasma has been used in dermatology [26] and wound healing applications in the medical field and in research; however, its use in dentistry is relatively new, with studies being carried out for sterilization (*in vitro*) of the root surfaces of the teeth, bleaching, or the removal of caries from the tooth. A user-friendly RC-plasma jet device capable of generating plasma within the root canal has been developed by Lu et al. [27]. The jet can be directly placed in the root canal, facilitating painless plasma disinfection. The device is currently undergoing clinical trials [28]. Additionally, Pierdzioch et al. [29] evaluated the effect of cold plasma on infected dentin both *in vitro* and ex vivo, demonstrating positive effects of plasma for the disinfection of dentin.

#### 2.1.1. Nonthermal Plasma as a Source of ROS Generation for Biomedical Applications

Plasma jets, corona discharges [30], and barrier discharges have been applied therapeutically [31]. Plasma jets can generate a column of plasma outside of electrodes into the surrounding area in a highly controlled manner, thus playing a crucial role in applications in the medical field. The ability of the plasma jet to penetrate into small structures via a direct or indirect mode as well as the small size and lightweight of this device makes it ideal for use during medical and dental treatments. The jet is operated such that there is a constant high voltage supply followed by the cutting off of the supply (plasma on and plasma off). This process helps to keep the temperature under control (less than 30°C) [31]. Nonthermal plasma (NTP) consists of partially ionized gases such as oxygen, helium, and argon. Low-pressure plasmas are known to generate very high concentrations of reactive species. Nonthermal plasma involves mostly electrons that are energized with no significant heating of ions or the constituent gases within [30].

**Figure 5:**
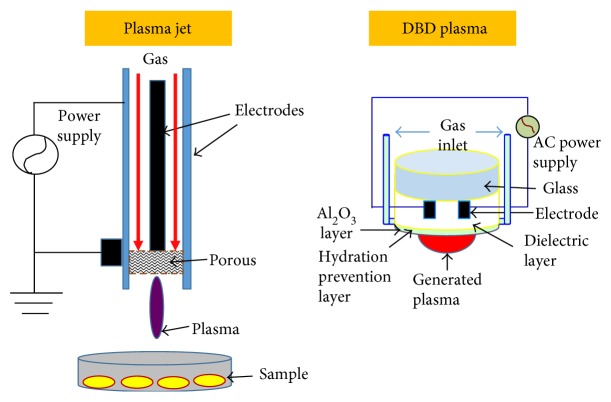
Components of a plasma jet and DBD (dielectric barrier discharge) plasma. A plasma jet can penetrate into small structures and it has a small size and lightweight, making it ideal for use in dental treatments. DBB plasma device consists of a carrier gas, which moves between two electrodes and is ionized to create plasma.

NTP has been used for the sterilization of instruments [32] and bacteria [33], in wound healing, and in other procedures (Figure 5). The argon plasma jet kINPen (a commercially available device) has been used successfully for wound healing procedures. When applied, the device generates ROS, ions, UV rays, magnetic fields, neutral particles, and others. However, if used correctly, these types of radiation are not harmful to humans. Variations of this device include kINPen 09 and kINPen MED (Leibniz Institute for Plasma Science and Technology-INP Greifswald and neoplas tools GmbH, Greifswald, Germany). The kINPen MED medical device is accredited for use on patients [34].

The introduction of nonthermal plasma in dentistry, due to its ease of use and painless characteristic, may eventually help to eliminate fear of dentistry in patients [35]. Nonthermal plasma can be used to treat various dental problems, such as the elimination of caries, root canal sterilization, and bleaching. The application of this type of plasma can be done by both direct and indirect means. O_3_, NO, and OH radicals are released, in biosolutions, as a result of interaction between plasma and liquid [36], which further act on cells and tissues. The use of nonthermal plasma for experimental [16] as well as clinical trials [34] has been done on a large scale in the medical field; however, in dentistry, clinical applications of nonthermal plasma are still in the nascent stage.

Dental biofilms are a major source of stress to clinicians, as they contain various types of bacteria, such as *Streptococcus* and *E. coli* [37]. A plasma application in conjunction with sprayed water has been used for the successful elimination of biofilms. Several researchers have confirmed that the use of a plasma device was superior to chlorhexidine for biofilm removal. Sun et al. [37] examined the effect of nonthermal plasma on gingival crevices, showing successful results. Jablonowski et al. [38] compared the effectiveness of an atmospheric pressure plasma jet (APPJ) with a sonic-powered brush for the removal of supragingival biofilm from extracted teeth. The biofilm removal outcomes in both cases were comparable, but the nonthermal plasma treatment resulted in additional chemical cleansing, leading to a significant plaque reduction. Schaudinn et al. [39] also developed a device for the elimination of biofilms on extracted teeth.

*Porphyromonas gingivalis*, a major periodontal pathogen, can be eliminated successfully with an intermittent plasma dose according to work by Mahasneh et al. [40]. Atmospheric plasma sources have also been used for alterations of surfaces for cell attachment for dental treatments such as the installation of titanium and poly-lactonic surfaces [41] and on dental biomaterials. Using plasma can increase the wettability of the implant surface, which facilitates the spreading of cells such as fibroblasts and osteoblasts, thereby enabling better osseointegration [42]. Chen et al. [43] found that treatment with a plasma brush can provide a super hydrophilic surface for enamel, dentin, and composites. The effect of a plasma treatment on the wettability of elastomeric impression materials was also investigated. Increased wettability results in a better impression of the tooth surface. Different materials such as addition silicones, condensation silicones, and polyether impression materials were used. It was concluded that the plasma treatment resulted in the formation of a high-energy impression surface on the tested materials [44]. Koban et al. [45] reported that the direct application of nonthermal plasma onto an untreated dentin surface reduced its contact angle, leading to better spreading of osteoblasts on the dentin. This may be utilized for future studies of periodontal regeneration.

Currently, several studies involving nonthermal plasma in dentistry are being carried out; however, due to the absence of standard protocols, comparison of results is very difficult. More studies of in vivo work in the dental field along with standardized protocols will facilitate the wider acceptance of nonthermal plasma in the dental field [46].

### 2.2. Composites and Resin Cements

Resin cements are used in dentistry on a large scale for the filling of cavities and for other restoration requirements resulting from cavities or abrasions. These cements remain in contact with the dentin-pulp complex for extended periods of time. Accordingly, their effects on pulp tissue should be assessed. Once polymerization takes place, monomers in the resin materials are released into the oral environment, with 2-hydroxy-ethyl methacrylate (HEMA) as a major constituent. The monomers can reach the pulpal tissue and cause irritation and hypersensitivity [47].

In a normal healthy cell, cellular reactions can result in the production of ROS, which is eliminated by antioxidants in the body. The mass production of ROS can result in cytotoxic effects and direct cell death. Certain resin monomers can leach out from the restorative materials in the oral cavity. TEGMA [47] is a major monomer that has been found as a leached byproduct of dental fillings. If TEGMA exceeds normal levels, it can attack the antioxidant system glutathione and deplete its levels, causing cell death. One study investigated an immortalized bovine dental papilla-derived cell line and an immortalized human dental papilla-derived cell line, which were analyzed for their cytotoxic reactions once exposed to resin cements (RelyX Unicem Clicker (RX), Max-Cem (MC), Panavia F 2.0 (PF), BisCem (BC), and Bistite II DC (BII)). The generation of ROS was found to occur in all the materials, but the lowest amount was with RelyX, making it a suitable material for use in dentistry [48]. Another study was done to assess the effect of dental resin monomers on dental pulp cells [49]. In that study, it was found that at higher concentrations, TEGDMA and HEMA inhibited the differentiation of dental pulp cells. That study confirmed that TEGMA and HEMA were responsible for activating the cell signaling pathways. Geurtsen et al. [50] also confirmed that TEGDMA and DPICI from light-cured glass ionomer cements or copolymers were the main causes of cytotoxic reactions and the release of free radicals, holding that the leaching of these materials should be minimized. More studies should be dedicated to uncovering how ROS particles are generated from cell cultures as a result of exposure to resin cements.

### 2.3. Lasers

Lasers have become popular in the healthcare industry with those who undertake hard and soft tissue procedures mainly due to their ease of usage and because they offer the lowest amounts of postoperative pain and sensitivity. Laser treatments in the dental field are carried out extensively during tissue repair [51] and gingivectomy procedures [52].

Low-level laser therapy (LLLT), also known as *cold laser therapy*, is a special type of laser therapy in which cells/tissues are exposed to very low levels of infrared light [53]. Upon the application of the laser therapy, reactive nitrogen and oxygen species are activated, which in turn activate the growth factors, TGF-*α*, TGF-*β*, and VEGF. This activity results in the proliferation and re-epithelialization of cells in the gingival and other tissues of the body at a faster rate with no discomfort or pain for patients [54]. In a study by Arany et al. [54], the pulp was exposed to laser-light-induced dental stem cells for tertiary dentine development, with successful results.

LLLT works through photoreceptor systems. Mitochondria are sensitive to near-infrared light (NIR). When exposed to laser therapy, mitochondrial photostimulation increases the level of ATP, with a subsequent transient increase in ROS levels as well [55]. ROS activation due to laser therapy, at low levels, causes the activation of growth factors and tissue repair processes [54], though occasionally, the excessive generation of ROS can arise, causing damage to the lipid and protein components of cells. However, depending on the intensity of the laser, the dose, the time of exposure, and other factors, defense mechanisms may be activated to check the excessive production of ROS [56].

### 2.4. Antimicrobial Photodynamic Antimicrobial Chemotherapy (PACT) and Dental Light Sources

**Figure 6:**
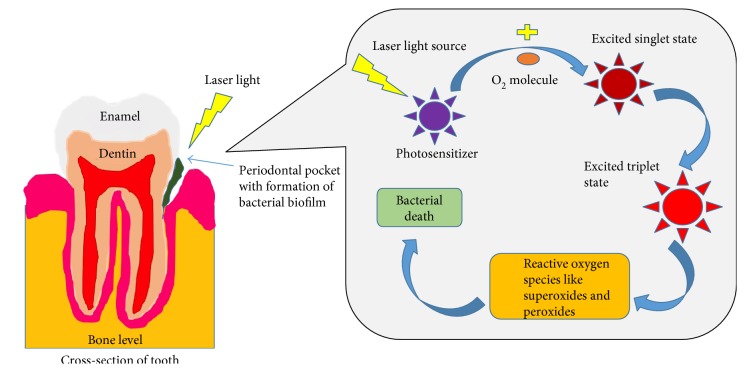
Mechanism of action of photodynamic antimicrobial chemotherapy (PACT). Photodynamic antimicrobial therapy is used as a periodontal treatment as an adjunct to scaling and root planing. When the laser or a light-emitting diode (LED) light source is used on the tooth/periodontal pocket, it causes a chain of events. The light source activates a photosensitizer, which reacts with oxygen to result in the formation of excited singlet oxygen (reactive species). They further form superoxides and peroxides which act on the bacteria and cause their elimination.

In the dental field, bacterial infections are a major source of distress to practitioners [57], and chemical disinfectants (like glutaraldehyde) may be carcinogenic at certain levels [58]. Accordingly, some of them are no longer used. Photodynamic antimicrobial chemotherapy (PACT) [59] uses light sources with narrowband wavelengths which activate a chemical that can produce ROS, such as H_2_O_2_ and ozone gas, possibly leading to cell death. This method is used for the elimination of root canal infections [59]. A variation of this is a type of antimicrobial photodynamic therapy (PDTa) used on cariogenic biofilms. The main molecule required during this process is oxygen for the generation of ROS. The method involves the generation of free radicals, which can occur by either of two processes. In the first process, the photosensitizer reacts with a substrate via an electron transfer and generates the radical species. In the second process, the photosensitizer reacts with oxygen to result in the formation of singlet oxygen (the reactive species) (Figure 6) [60]. This therapy has been used as a periodontal treatment as an adjunct to scaling and root planing. In vitro studies, for the treatment of bacteria [61, 62], have been conducted on a large scale, but there is no significant study of the increased level of clinical attachment apart from one study by Andersen et al. [63]. They found that photodynamic therapy along with root planing/scaling resulted in better clinical attachment levels, less bleeding upon probing, and less gingival recession. It was shown by Spagnuolo et al. [64] that adhesive materials produce intracellular ROS depending on the material and the type of light source used.

The OptiBond adhesive system shows low toxicity when treated with a halogen light source, and the Scotch bond system when treated with a halogen lamp or LEDs produces high amounts of ROS. Visible light, especially blue light (400–500 nm), is widely used in dentistry for the curing of resin cements on the tooth structure. The extent of irradiation onto oral tissues is not clear, though it may cause cellular damage given its high energy and good penetration depth within cells and tissues [65]. Yoshida et al. [66] showed that blue light induced vasoconstriction in rat aortas due to the generation of reactive oxygen species. They also reported that irradiation with blue light causes the generation of ROS within gingival fibroblasts. Blue light induces ROS generation and results in vasoconstriction, and recovery from vasoconstriction also causes ROS generation (the “vicious circle” effect), which can affect the pulp and cause it to age [66].

### 2.5. Bleaching Agents and Intracanal Medicaments

Bleaching agents result in tooth whitening given their use of various oxidizing agents. Hydrogen peroxide, sodium hypochlorite, and ozone are the agents most commonly used. They generate ROS which may penetrate through the dentin and cause the decomposition of organic materials. The exact mechanism of the bleaching of teeth using peroxides is unknown.

A study was conducted by Eimar et al. [67] to identify the mechanism of bleaching. They concluded that treating the enamel with hydrogen peroxide was more effective than other mechanisms (i.e., NaOH, EDTA cleansing) and that the peroxide released free radicals that acted on the organic component of the enamel due to an oxidative process. Saita et al. [68] found that TiO_2_ coated with hydroxyapatite can generate ROS to result in superior bleaching effects on samples. This could be a new clinical application for dental bleaching.

However, the use of hydrogen peroxide (a common bleaching agent) may also have harmful effects on soft and hard oral tissues. Lee et al. [69] studied the influence of H_2_O_2_ on odontoblasts, and with 0.3 mmol/L of H_2_O_2_, ROS accumulation was detected within the cells, which facilitated cell differentiation. It can be inferred that ROS at low amounts can produce favorable results, though at high concentrations, it may bring harm to cells [69].

Endodontic therapy requires the use of various intracanal medicaments for the disinfection of root canal systems. The use of medicaments such as sodium hypochlorite, calcium hydroxide, and chlorhexidine is very common for root canal procedures. In vitro studies have demonstrated that 0.2% chlorhexidine may induce ROS formation when mixed with Ca(OH)_2_ within a few days [70]. The accumulation of ROS intermediates can contribute to the destruction of root pathogens [70] or to bone loss and periapical tissue degradation depending on the amount of chlorhexidine used [71].

### 2.6. ROS from Ionizing Radiation and Ultraviolet Rays

Ionizing radiation is electromagnetic radiation which is capable of removing electrons from atoms. These are of two types: photon (X-rays and gamma rays) and particle (alpha particles, beta particles, and neutrons) ionizing radiation. X-rays and gamma rays are known to be human carcinogens [72]. Cells can undergo radiation damage directly (damage to DNA molecules) or indirectly (the generation of free radicals), resulting in physiological and biological alterations. Ionizing radiation attacks water (a major constituent of cells) and organic molecules, causing hydroxyl (OH•), alkoxy (RO), and other free radicals to be released. These free radicals are toxic to the DNA molecular structure [73].

The generation of ionizing radiation is a common phenomenon in a dental setup. Radiation exposure may occur as a result of head and neck cancer treatment and/or radiotherapy. Radiographic examinations are also an indispensable aspect of dental practice [74]. The detection of caries, evaluations of periodontal disease and bone loss, overhanging restorations, and cone beam-computed tomography (CBCT) for the assessment of dental implants placed in the mouth at accurate positions all require some amount of radiation exposure. The dental practitioner should be aware of the diagnostic reference levels (DRL), which act as guide to understand if patient exposure has occurred [74]. Dental radiographic selection criteria have long been established, but compliance remains low. Dentists should be well informed about the various types of equipment and techniques which can reduce patient exposure and enhance the capability of radiography as a tool.

UV rays in dentistry can be found in a variety of procedures. The UV spectrum is classified as vacuum UV (40–190 nm), far UV (190–220 nm), UVC (220–290 nm), UVB (290–320), and UVA (320–400 nm) [75]. Some amount of UV is beneficial for the human body, as it stimulates the production of vitamin D within the body and can be used as a type of phototherapy in certain medical cases (immune therapy). However, at high levels, it can also generate significant amounts of free radicals and thus result in skin ageing and even cancer. In dentistry, the detection and identification of resin cement and/or its complete removal can be done by UV illumination [76]. Germicidal lamps for the identification and killing of bacteria [77] and for sterilization have been successfully used. Ultraviolet photofunctionalization is a new mode of surface treatment for dental implants. Various studies have been carried out in this area [78–80]. In the typical procedure, surface contaminants (hydrocarbons) are removed by decomposition and photocatalysis through UV rays [81]. This helps to improve the wettability of the implant surface and results in more cases of successful osseointegration [82].

## 3. Effects of ROS on Dental Cells

The use of nonthermal plasma has recently become popular in the medical and dental fields. This type of plasma can be used for cell sterilization, can be applied to living tissue directly, and has been utilized for wound healing and angiogenesis [83]. In dentistry, with the advent of the osseointegration concept, the surfaces of titanium [84, 85] and zirconia (implant materials) have been treated with nonthermal plasma and analyzed extensively. This type of plasma can also induce polymerization [86], but its direct effect on stem cells in the mouth cavity should be studied. Research has started in this field [37, 39–41]; however, it is still relatively a new field to explore. Cold atmospheric pressure has been successfully analyzed for the treatment of cancer cells within the human body. The action affects the mitochondria, ROS, and RNS species, which can affect signaling mechanisms to induce apoptosis with the arrest of cells within the S-phase [87]. The effect of low-temperature nonthermal plasma on PDLSC-derived mesenchymal cells was analyzed by Miletic et al. [88] in what was at the time a novel study of the use of a plasma technique for periodontal therapy. There were no effects on the viability of cells, but increased osteogenic differentiation was noted with a direct plasma treatment. Living tissues were analyzed after being exposed to cold atmospheric pressure by Shashurin et al. [89]. They suggested an intense treatment with cold plasma, though a direct impact too close to cells can result in their death by desiccation.

With a decrease in the level of treatment from moderate to mild, detachment and the cell migration rate are also reduced [89]. The removal of cells using cold plasma may be utilized in the future for selective tissue removal. Kieft et al. [90] deduced that a plasma treatment can cause the slow onset of apoptosis as many as 16–24 hours later compared to other inducers of cell death (the timing of apoptosis depends on the cell type). As noted above, free radicals (RNS/ROS) are like a double-edged sword [91]. At low levels, they show positive effects such as cell detachment and the effective proliferation of bone cells, but they have been also linked to degenerative procedures such as ageing and death [92], though this has been nullified by many researchers. Free radicals are not a cause of ageing but still may be related to it. Gas plasma technology has been used to modify polylactic acid surfaces to enhance the osteogenic differentiation of MC3T3-E1 cells. NO has been shown to play an important role in the differentiation of osteoblasts [92].

Dobrynin et al. [93] showed that plasma can have an effect on intracellular calcium, resulting in a slow increase, and that antioxidants can suppress the effects of plasma. Head and neck cancer (squamous cell carcinoma) require a combination of surgery and radio and chemotherapy, which may not be 100% successful. Preston et al. [94] used CAP successfully on HNSCC (head and neck squamous cell carcinoma) cell lines and showed that in a dose-dependent manner, the viability of HNSCC was diminished by CAP. A similar study was done by Welz et al. [95], showing apoptosis of HNSCC cell lines using CAP. Delben et al. [96] used cold plasma on the *in vitro*-reconstituted oral epithelium (oral keratinocytes cultured on collagen-based matrix) EpiOral™ and indicated no significant damage to cells with low toxicity and high viability levels.

Matsui et al. [97] researched the effect of reactive oxygen species on the calcification ability of human dental pulp cells using a Ga-Al-As laser with successful results. It was confirmed that ROS can induce cell differentiation in vitro and that irrigating the pulp with H_2_O_2_ may be beneficial for dentinogenesis.

## 4. Conclusion

The roles of ROS within the human body must be analyzed fully. They play key roles in cell signaling pathways and redox reactions. At low concentrations, they can be highly beneficial for processes such as tissue repair and angiogenesis, while at high concentrations, tissue injury and/or apoptosis may occur.

The various sources of ROS within the dental field, such as nonthermal plasma and laser bleaching agents, can have positive and negative impacts on the human body. Periodontitis is a common infectious disease encountered by dental practitioners. Nonthermal plasma and lasers based on the principles of ROS can be utilized to eliminate periodontal pathogens. A considerable amount of research has been undertaken in this arena.

Stem cell therapy has emerged as a new treatment option for reinstating cells and tissues, and efforts are being made to use this treatment modality in periodontal therapies. The effect of nonthermal plasma in the dental field is a new aspect, and the interactions between plasma-generated species and dental stem cells have still not been explored, as this is a relatively new field with a broad scope for research. As of now, very little is known about the effect of nonthermal plasma on mesenchymal and other dental stem cells [88] and understanding the pathological and therapeutic effects of free reactive species clearly with different parameters is one of the major focuses of the research for bright future prospects of this technology [91]. Efforts should be made to increase research on the interaction of ROS species with various dental stem cells for more rapid bone formation and healing [98]. Although the biological significance of these procedures is yet to be established, they may be useful in the future for cellular-based therapies [99].

## Figures and Tables

**Table 1:** ROS regulation of pathways.

Parameter	Cellular pathways	Enzymes involved	References
Exogenous or endogenous ROS	Antioxidant, anti-inflammatory response	Nrf2 Ref1	[16, 17]
	DNA damage	ATM	[16, 18]
	Iron hemostasis	IRP	[16, 17]
	Cellular proliferation, survival, differentiation	ASK1, PI3K, PTP, Shc	[16–18]

Nrf2: nuclear factor erythroid 2- (NFE2-) related factor 2; Ref1: redox-factor 1; ATM: ataxia-telangiectasia mutated; IRP: iron regulatory protein; ASK1: apoptosis signal-regulated kinase 1; PI3K: PI3 kinase; PTP: protein tyrosine phosphatase; Shc: Src homology 2 domain containing.

**Table 2:** Sources of ROS in dentistry.

ROS sources in dentistry	Notes	References
(1) Application of nonthermal plasma	RC plasma jet for root disinfection	[25]
	Cold plasma effect on dentin	[27]
	Plasma jets generate ROS	[28, 29]
	Sterilization of instruments	[30]
	kINPen device for wound healing	[32]
	Release of hydroxyl and other ions in biosolutions	[34]
	Removal of bacteria in gingival crevices	[35]
	APPJ for biofilm removal	[36, 37]
	*P. gingivalis* elimination with plasma	[38]
	Plasma results in changes in surface texture of dental implants	[40, 41]
	Cleaning of dentin for better periodontal regeneration	[43]

(2) Composites and resin cements	Monomers (after polymerization) irritate pulpal tissue and release ROS	[45–47]
	TEGMA/DPICI from GIC (light cured) release free radicals	[48]

(3) Laser	Tissue repair	[49]
	Gingivectomy	[50]
	LLLT causes ROS release by mitochondrial photostimulation	[52–54]

(4) Photodynamic therapy and light sources	PACT produces ROS, H_2_O_2_, ozone gas	[57]
	PDTa on carcinogenic biofilms releases ROS	[62]
	Adhesive materials produce ROS	[63]
	Visible light (400–500 nm) can release ROS and cause cellular damage	[63, 64]

(5) Bleaching agents and intracanal medicaments	EDTA and NaOH release free radicals that act on enamel	[65]
	TiO_2_ coated with hydroxyapatite release ROS	[66]
	Influence of H_2_O_2_ on odontoblasts	[67]
	Chlorhexidine + Ca(OH)_2_ induces ROS that destroy root pathogens	[68, 69]

(6) ROS from ionizing radiation and UV rays	Ionizing radiation release free radicals, harmful to DNA molecule	[71]
	CBCT, radiotherapy, periapical X-ray common in dentistry	[72]
	UV rays for skin treatment, elimination of cancer	[73, 74]
	Photocatalysis through UV rays	[79]

APPJ: atmospheric pressure plasma jet; *P. gingivalis*: *Porphyromonas gingivalis*; ROS: reactive oxygen species; TEGMA: triethylene glycol dimethacrylate; DPICI: diphenyliodonium chloride; LLLT: low-level laser therapy; PACT: photodynamic antimicrobial chemotherapy; PDTa: antimicrobial photodynamic therapy.
